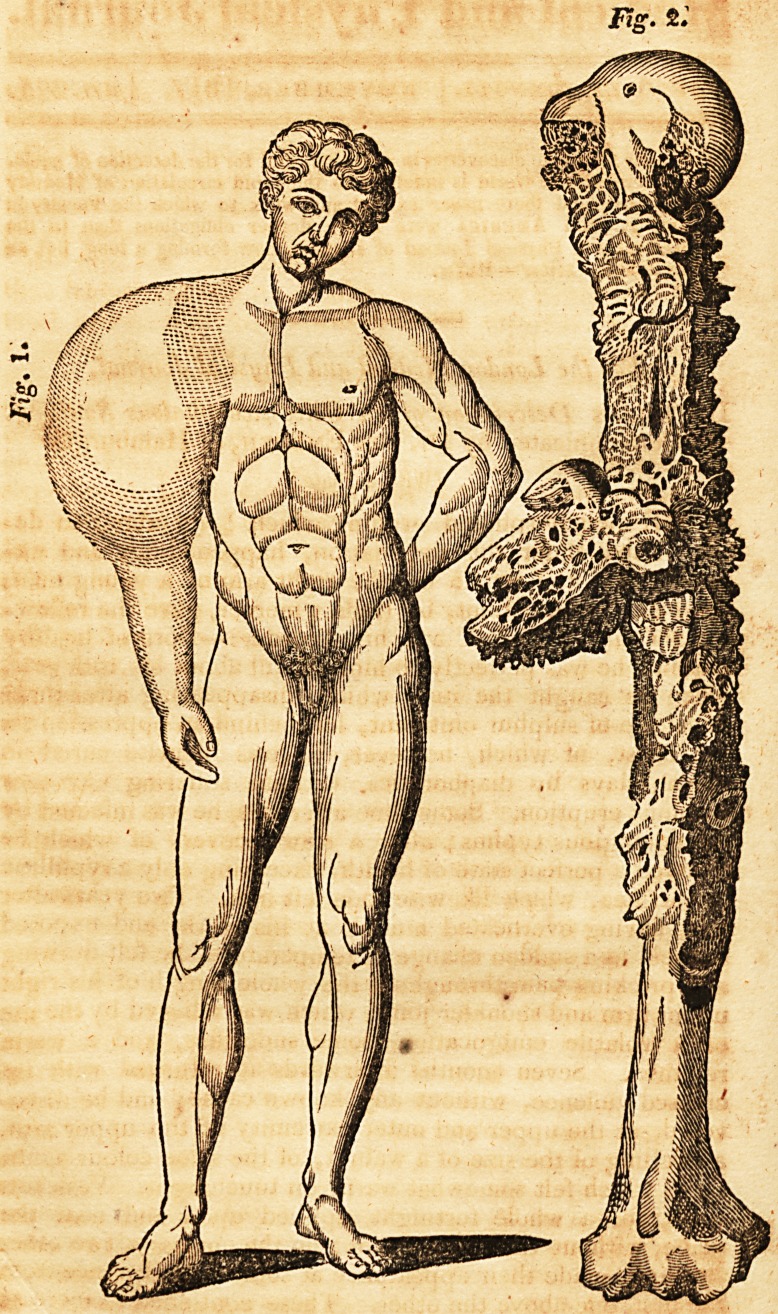# Dr. Rust's Description of an Osteo-Steatomatous Swelling

**Published:** 1817-11

**Authors:** 

**Affiliations:** of Hamburgh.


THE LONDON

### Medical and Physical Journal.

5 OF VOL. XXXVIII.] NOVEMBER, 1817. [NO. 225.

" For many fortunate discoveries in medicine, and for the detection of numerous errors, the world is indebted to the rapid circulation of Monthly Journals; and there never existed any work to which the Faculty in Europe and America were under deeper obligations than to the Medical and Physical Journal of London, now forming a long, but an invaluable, series."—Rush.

*For the London Medical and Physical Journal.*

### Dr. Rust's *Description of an Osteo-steatomatous Swelling:* Communicated by Dr. von Embden, of Hamburgh.

(With a Plate.)

THE metamorphosed swelling which I am about to describe under this appellation, happened in, and extended over the whole of, the right arm of a young man, aged 23. The patient, by trade a mercer, gave the following account of himself and his disorder:—Born of healthy parents, he was perfectly so himself, till about his 19th year, when he caught the itch, which, disappearing after three days' use of sulphur ointment, left behind an oppression on the chest, of which, however, he was likewise cured in eleven days by diaphoretics, without suffering any new scabious eruption. Some time after this, he was infected by the contagious typhus; after a slow recovery of which he enjoyed a perfect state of health, excepting only a syphilitic gonorrhœa, which likewise soon left him. Two years after this, having overheated himself at his work, and exposed himself to a sudden change of temperature, he felt drawing and pricking pain throughout the whole length of his right upper arm and shoulder joint, which was relieved by the use of a volatile embrocation, some sudorifics, and a warm regimen. Seven months afterwards it returned with increased violence, without any known cause; and he discovered, at the upper and outer extremity of the upper arm, a swelling of the size of a walnut, of the same colour as the skin, which felt somewhat warm on touching it. Vesicants were, for a whole fortnight, applied upon and near the place, without the least benefit: on the contrary, two other swellings made their appearance at some little distance from the first, one above the other. These continued to increase till they appeared to unite. Tormented with pain and watch-

 fulness, fulness, and declining in health every day, the patient applied every-where, even to the Hospital of Charitable Brethren at Vienna, for relief, but found none. All sorts of remedies, including warm sulphureous baths and electricity, were tried without the least advantage. The electric sparks, which had been carried right through the swelling, he thought, had increased the tumour. The powders also (mercurial as it should seem) had induced salivation, but no amendment.

He now left the hospital, after six weeks, in a worse state than he had entered it, spent another three weeks under the most grievous domestic troubles, the disorder rapidly increasing upon him; till at last, in December 1812, he resolved to seek relief at the practical school of Prof. R., who, declaring the disorder to be a *metamorphosis sui generis*, ordered him fomentations of arnica and mint, and sent him away. Disappointed and out of patience by the fruitless experiments for its dispersion, for five whole months, and tired of prolonging his existence with unavailing palliatives, he thought fit to apply to me for advice, and caused himself to be carried to my division in the General Infirmary, firmly resolved to undergo the operation. On first seeing the patient, the disorder described by Marcus Aurelius Severinus, in his work De Abscondita Abscessuum Natura (Lugd. Batav. 1724, page 207), seemed to present itself: only that the swelling in the present case was more protuberant in front, and not of so great a bulk. The patient, who bore evident marks of a scrofulous diathesis, was rather of an active and irritable habit; yet declining energy was plainly expressed in his dull tearful eyes and emaciated muscle, by his continued febrile motions easily excited, by his perspiration, want of appetite, and great debility.

On the affected upper arm there appeared a swelling extending, not sharply limited, from the spina scapulæ over the shoulder-joint, in front, towards the clavicula, approaching in figure the shape of an inverted pyramid, with a spherical basis, the pointed part of which is turned downwards towards the fore-arm, but the basis upwards,—a little protuberant, however, near the detoid muscle. Its largest circumference, from the clavicula over the larger tubercle to the spina scapulæ, was fifteen Vienna inches and a half; and the smallest, over the condyle of the upper arm, six inches. Besides this, the whole fore-arm was œdematous down to the finger-points; the surface of the swelling was of a pale red, yet darker in some places, and had livid spots of various sizes; varicose vessels ran through the integuments in various directions, and gave the swelling a marbled appearance.

appearance. When touched, it felt like a firm substance, though not all over of the same solidity; a fluctuation of a deep-seated fluid could not, nevertheless, escape the feel of the experienced surgeon, though the skin was very tight. The temperature of the swelling was scarcely above the normal degree; its weight was so considerable, that it drew the whole carriage of the body towards the right side, and that the motion of the body was in part governed by it. (See the Plate, fig. 1.) The motion of the part affected, though much confined on account of the tension and swelling, neither encreased the pain, nor was it attended with creaking and cracking in the shoulder-joint. The pain was in general considerable and tearing, principally deeply seated, extending to the finger-points, and of such continuance as to allow him not a moment's reprieve or sleep; so that, tired of his sufferings, he prayed for relief by either cure or death.

As far as I know, besides the above-mentioned case of Marc. Aur. Severinus, there is but one observation on record, which, though different with regard to the early symptoms, is very similar to the present in respect to its course, viz. that mentioned by Böyer in the 5th chap. of the 2d vol. of his "Leçons sur les Maladies des Os, à Paris, 1803."

I consulted several eminent physicians and surgeons, partly on account of its rareness, and partly on account of the importance of the case, among whom were the Professors Zang and Raiman, and Mr. von Zellenbergh, head-surgeon. All the phenomena, in conjunction with those emanating from the early symptoms, were, indeed, sufficient to satisfy me about the impossibility of effecting a cure by either dry, wet, or pharmaceutical remedies. But we were not at all nearer a satisfactory diagnosis, as the disorder might as well have originated from a scrofulous, scabious, rheumatic, arthritic, and syphilitic cause, as from a purely local unknown one; and, as to form, might belong as well to the osteo-sarcomatous, steatomatous, carious, arterious, venous, lymphatic, or any other sort of metamorphosis,—nay might, perhaps, be a combination of them all at the same time. Prof. K. was not, indeed, so much in the wrong in declaring it to be a *metamorphosis of a peculiar kind;* yet it is to be regretted that these denominations signify so little, as that they express little more than nothing. It could not be doubted that the disorder at this time was in the bone, as well as the softer parts, which was evident as well from the former and present phenomena, as from the observations of other writers on similar diseases; but whether the primitive affection was to be looked for in the muscular, cellular, tendinous, or vascular structure—what might truly be the cause and what the the effect—was beyond our surmise; nor did, in my opinion, even the dissection of the arm afterwards give a satisfactory or thoroughly decisive insight on that head.

Without intending to speculate on the primitive or subsequent formation of the disease, I describe what I saw, leaving it to those who may think themselves superior judges, without being even acquainted with the individual case, to accuse me of forming a fallacious diagnosis, and even impute the bad success of the operation to my deficiency in fixing the diagnosis. To myself and the gentlemen present, it was enough to know, that nothing less than an operation could save the patient; and that, unless it was soon put in execution, death would overtake him; and that, by the operation, (which, indeed, he wished for himself,) there might possibly a great deal be gained, though, in the worst case, nothing could be lost by it. That the amputation of the upper arm from the shoulder-joint was practicable, and even indicated (a point disputed by that party), I was led to conclude, partly from the circumference and extension of the affection, and partly from the most apparent certainty that, according to every symptom, the morbid metamorphosis had not yet taken hold of the glenoid cavity. It was, however, the general opinion, that an incision should be made in the swelling previous to our proceeding to the extirpation, in order to fix upon a diagnosis, and then to regulate our further measures accordingly. An assistant, therefore, being placed so as to compress the subclavian with his thumb, I made an incision about two inches long, and one and a half deep, on the inner side of the upper arm, in that place where, in a natural state, the brachial artery has its course. A steatomatous scirrhous substance now appeared; on examining which with my fingers, and penetrating deeper with it, I reached a cavity, from which, on withdrawing my finger, issued forth uninterruptedly a large quantity of clear blood. We now thought to have found an aneurismal sac, and, there being neither room to think of finding and tying the artery in so degenerate a state of all parts, nor of losing so much time by the incessant flow of the blood from the aperture that had been made, I instantly proceeded to the amputation; in doing which, I was unable to follow the rules laid down for the cut in the schools for practical surgery; for, though they are generally founded upon precise anatomical principles, yet they are solely calculated according to the normal formation of the parts; whereas I was obliged to pay particular attention to the morbid formation of the neighbouring soft parts, a point which has been too much disregarded by writers on surgical operations. Ligatures were instantly applied; applied; and, though all this was done as quick as possible, the patient was, nevertheless, so much debilitated by the loss of blood sustained during the operation, that he fainted away at the end of it. He was, therefore, put to bed immediately afterwards; and, the healthy state of the acromion being ascertained, an easy bandage, such as fitted the purpose, was applied.

The patient soon recovered from his swoon, and gave hopes of future amendment. Long previous sufferings had, however, brought him so low, that his debility could not be conquered, and he died of it on the fourth day from the operation. The only thing I have to regret in this case is my not having instantly undertaken the amputation, without previously making an incision, as it would have spared the patient a considerable loss of blood, and, as I am led to think from subsequent examinations, he probably then might have been saved by it. In cases like the present, we are generally only prepared to meet with arterious hæmorrhages; but it is almost incredible how great a quantity of blood flows from the varicous distended veins, when cut into, in such-like metamorphoses. I therefore consider it a duty incumbent on myself to direct the attention of junior practitioners to this circumstance, for, proper as it is in similar dubious cases to make an incision in the swelling in order to ascertain the diagnosis, and to conduct the operation accordingly, or, if possible, to spare the limb, yet too great care and caution in the operator may prove as dentrimental to the patient as too much boldness and carelessness.

The dissection of the enucleated arm shewed a perfect degeneration of the softer parts of two-thirds the superior of the fore-arm, which were thoroughly penetrated by new vessels. No trace of true cellular or muscular fibre was to be seen. The artery which was to be searched after in the fore-arm, and followed up in front, ran right through the steatomatous and cartilaginous substance, and, to our great surprise, was found entire and sound. The degeneration of the other soft parts penetrated, in many places, to the bone, and, on trying to separate them from it, a swelling as big as a fist was found at the anterior, inner, and outer surface of the upper arm, distinct, by its hardness, from the other degenerated parts, joined to the bone by a ligamentous connexion,—in which ossification had already advanced so far that even maceration could no longer destroy it. In other places, where the degeneration of the softer parts had not penetrated to the bone, cavities filled with blood had formed themselves. The bone itself, which is preserved in the Pathological Museum of the Infirmary at Vienna, was entirely destroyed destroyed by caries, from the neck of the condyle down to the last third part of it, and, in one place, is so much destroyed by the worm-eaten caries, that a probe could be passed through it into the medullary cavity; but the condyle itself and the acromium were still covered with a perfectly healthy cartilage. (See the Plate, Fig. 2.)

---

*For the London Medical and Physical Journal.*

## Figures and Tables

**Fig. 1. Fig. 2. f1:**